# The circadian gene ARNTL2 promotes nasopharyngeal carcinoma invasiveness and metastasis through suppressing AMOTL2-LATS-YAP pathway

**DOI:** 10.1038/s41419-024-06860-x

**Published:** 2024-07-02

**Authors:** Wenqing Zou, Yiming Lei, Cong Ding, Hongjun Xiao, Shunxin Wang, Shaobo Liang, Weijie Luo, Zhiqing Long, Shiwei He, Qingjie Li, Han Qiao, Na Liu, Yanping Mao

**Affiliations:** 1 Department of Radiation Oncology, Sun Yat-sen University Cancer Center, State Key Laboratory of Oncology in South China, Collaborative Innovation Center of Cancer Medicine, Guangdong Key Laboratory of Nasopharyngeal Carcinoma Diagnosis and Therapy, Guangzhou, China; 2 Department of Otorhinolaryngology, Union Hospital, Tongji Medical College, Huazhong University of Science and Technology, Wuhan, China; 3 Department of Radiation Oncology, The Third Affiliated Hospital of Sun Yat-Sen University, Guangzhou, China; 4 Department of Medical Oncology, The Seventh Affiliated Hospital of Sun Yat-sen University, Shenzhen, China

**Keywords:** Oncogenesis, Metastasis

## Abstract

Metastasis is the major culprit of treatment failure in nasopharyngeal carcinoma (NPC). Aryl hydrocarbon receptor nuclear translocator like 2 (ARNTL2), a core circadian gene, plays a crucial role in the development of various tumors. Nevertheless, the biological role and mechanism of ARNTL2 are not fully elucidated in NPC. In this study, ARNTL2 expression was significantly upregulated in NPC tissues and cells. Overexpression of ARNTL2 facilitated NPC cell migration and invasion abilities, while inhibition of ARNTL2 in similarly treated cells blunted migration and invasion abilities in vitro. Consistently, in vivo xenograft tumor models revealed that ARNTL2 silencing reduced nude mice inguinal lymph node and lung metastases, as well as tumor growth. Mechanistically, ARNTL2 negatively regulated the transcription expression of AMOTL2 by directly binding to the AMOTL2 promoter, thus reducing the recruitment and stabilization of AMOTL2 to LATS1/2 kinases, which strengthened YAP nuclear translocation by suppressing LATS-dependent YAP phosphorylation. Inhibition of AMOTL2 counteracted the effects of ARNTL2 knockdown on NPC cell migration and invasion abilities. These findings suggest that ARNTL2 may be a promising therapeutic target to combat NPC metastasis and further supports the crucial roles of circadian genes in cancer development.

## Introduction

Nasopharyngeal carcinoma (NPC) is a metastasis-prone head and neck malignancy [1, 2]. As reported, over 70% of patients develop metastasis to cervical lymph nodes [2, 3], and 20–30% of cases experience distant metastasis, most commonly to the bones, lungs, and liver [4]. Modern clinical practice has largely improved the disease progression of NPC patients owing to early screening and improved therapeutic regiments, such as plasma Epstein-Barr virus DNA detection and the combination of intensity-modulated radiotherapy and chemotherapy [5, 6]. However, the survival benefits of patients with metastasis remain very limited. Therefore, it is extremely urgent to elucidate the molecular mechanism involved in NPC metastasis to facilitate the development of specific therapeutic options against NPC metastasis.

Circadian rhythms, driven by circadian genes, are widely present in almost all organisms and coordinate various biological processes [7]. Epidemiological evidence supports that disruption of circadian rhythms increases the risk of human cancers, including nasopharyngeal carcinoma, colon carcinoma, breast cancer, prostate cancer, and lung cancer [8–13]. As a result, “shift work that involves circadian disruption” is classified as potentially carcinogenic to human (Group 2A) by the International Agency for Research on Cancer (IARC) in 2007. These data suggest that dysregulation of circadian genes is potentially important in the pathogenesis of cancers. Recent studies have reported that multiple circadian genes, especially PER-ARNT-SIM (PAS) superfamily, are responsible for NPC progression. For example, overexpression of PER2 attenuated NPC cell proliferation, metastasis, and chemoradiotherapy resistance [14]. Additionally, our colleagues’ previous study revealed that ARNTL exhibited low expression in NPC and inhibited tumorigenesis and cisplatin resistance by targeting CDK5, but did not affect cell migration and invasion abilities [15]. Interestingly, we found that the circadian transcription factor ARNTL2, a paralog of ARNTL, was upregulated in NPC when we analyzed GSE12452 dataset, indicating that ARNTL2 may have different roles from ARNTL in the development of NPC. However, the role and mechanism of ARNTL2 in NPC are yet poorly understood.

In this study, we identified that a core circadian gene, ARNTL2, played an essential role in NPC metastasis. Our results showed that the upregulation of ARNTL2 significantly facilitated NPC cell migration and invasion abilities by inhibiting AMOTL2 transcription to increase YAP nuclear translocation. The findings give sights into the role and mechanism of ARNTL2 in NPC.

## Results

### ARNTL2 is highly expressed in NPC tissues and cells

**Fig. 1:**
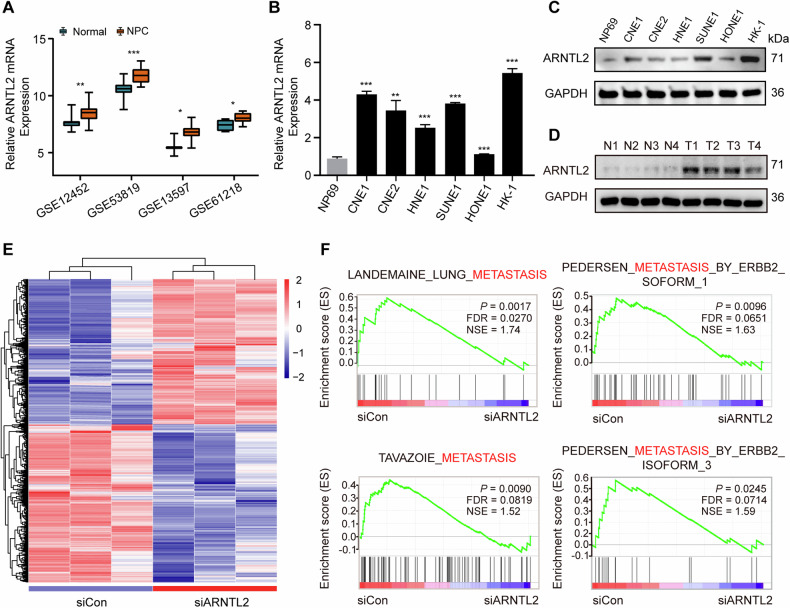
ARNTL2 is upregulated and associated with metastasis in NPC. **A** The relative mRNA levels of ARNTL2 in NPC tissues and normal tissues based on four microarray data from the GEO database (GSE12452, GSE53819, GSE13597, and GSE61218). **B**, **C** The relative mRNA and protein levels of ARNTL2 in a normal nasopharyngeal epithelial cell line (NP69) and six NPC cell lines (CNE1, CNE2, HNE1, SUNE1, HONE1, HK-1) followed by RT-qPCR and western blotting. **D** The protein level of ARNTL2 in normal tissues and NPC tissues. **E** Heatmap of the differentially expressed genes (|fold-change| > 1.5 and *P* < 0.05) from the RNA seq data based on HONE1 cells with or without ARNTL2 knockdown (*n* = 3). **F** GSEA analysis of the RNA-seq data revealed that high expression of ARNTL2 was correlated with tumor metastasis. Data are presented as means ± SD. *P < 0.05; **P < 0.01; ***P < 0.001.

To gain an insight into the expression status of ARNTL2 in NPC, we first downloaded four public NPC microarray data (GSE12452, GSE53819, GSE13597, and GSE61218) and compared the mRNA level of ARNTL2 between NPC and normal tissue samples. The results showed that the mRNA level of ARNTL2 in NPC tissues was dramatically upregulated compared with normal tissues (Fig. 1A). Also, we evaluated the mRNA levels of ARNTL2 in a normal nasopharyngeal epithelial (NPE) cell line (NP69) and six NPC cell lines (CNE1, CNE2, HNE1, SUNE1, HONE1, and HK-1). The results also confirmed that the mRNA level of ARNTL2 in NPC cells was significantly higher (Fig. 1B). Subsequently, we detected the protein levels of ARNTL2 in NPE and NPC tissues and cells, and obtained consistent results (Fig. 1C, D). These data suggest that ARNTL2 is highly expressed in NPC and might function as an oncogene in tumor progression.

### ARNTL2 promotes NPC cell migration and invasion abilities in vitro

To investigate the potential biological role of ARNTL2 in NPC, we performed RNA-seq analysis in HONE1 cells with or without ARNTL2 knockdown. As shown in Fig. 1E and Supplementary Table S3, a total of 1623 differentially expressed genes, including 777 upregulated and 846 downregulated genes, were identified (|fold-change| > 1.5 and *P* < 0.05). GSEA analysis revealed a significant enrichment of multiple metastasis-related gene sets in the high ARNTL2 expression group (Fig. 1F), indicating potentially a pro-metastatic role of ARNTL2 in NPC.

**Fig. 2:**
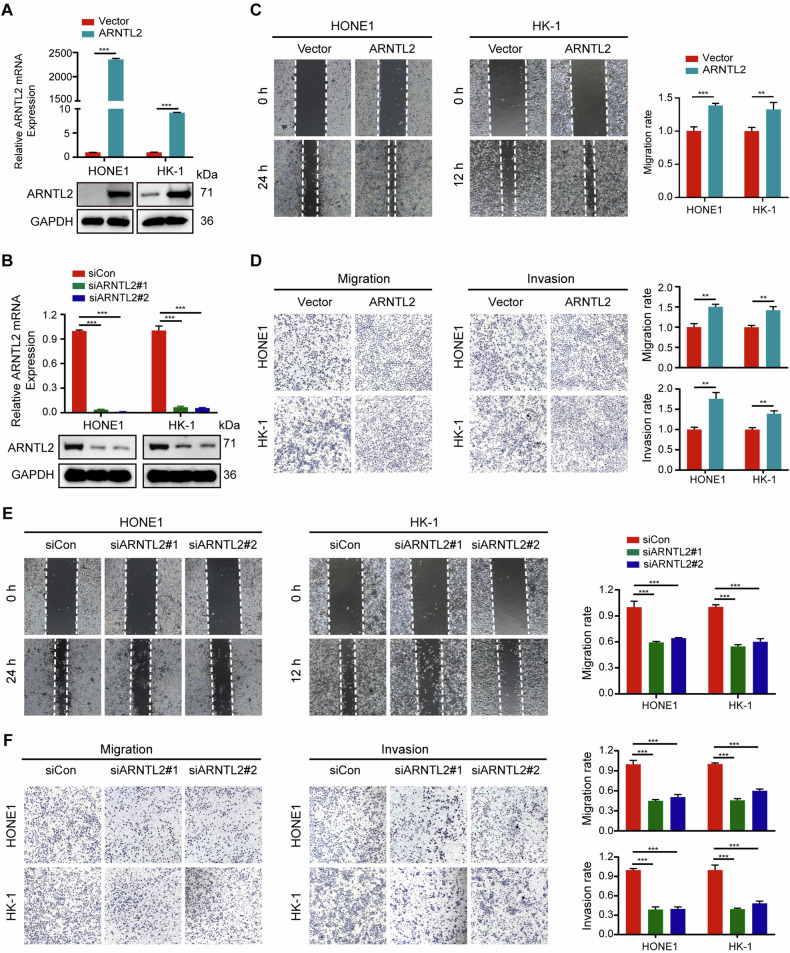
ARNTL2 promotes NPC cell migration and invasion abilities in vitro. **A**, **B** The transfection efficiencies of ARNTL2 overexpression and knockdown in HONE1 and HK-1 cells using RT-qPCR and western blotting assays. **C** Wound healing migration assay in HONE1 and HK-1 cells with ARNTL2 overexpression (× 100). **D** Transwell migration and invasion assays without or with Matrigel in HONE1 and HK-1 cells with ARNTL2 overexpression (×100). **E** Wound healing migration assay in HONE1 and HK-1 cells with ARNTL2 knockdown (×100). **F** Transwell migration and invasion assays without or with Matrigel in HONE1 and HK-1 cells with ARNTL2 silencing (×100). Representative images are left panel and statistical analyses are right panel. Data are presented as means ± SD. **P < 0.01; ***P < 0.001.

To confirm whether ARNTL2 is a regulator of metastatic ability in NPC, we performed transient overexpression or knockdown of ARNTL2 in EBV-negative (HONE1) and EBV-positive (HK-1) NPC cell lines (Fig. 2A, B). Wound healing and Transwell assays were then performed to explore the effects of ARNTL2 on NPC cell migration and invasion abilities. Notably, overexpression of ARNTL2 enhanced NPC cell migration and invasion abilities (Fig. 2C, D). In contrast, knockdown of ARNTL2 remarkably impaired NPC cell migration and invasion abilities (Fig. 2E, F). However, neither overexpression nor depletion of ARNTL2 had no or little effect on NPC cell proliferation (Supplementary Fig. S1). These findings indicate that ARNTL2 may mainly promotes metastasis in NPC development.

### ARNTL2 represses the expression of AMOTL2 by regulating its transcription

**Fig. 3:**
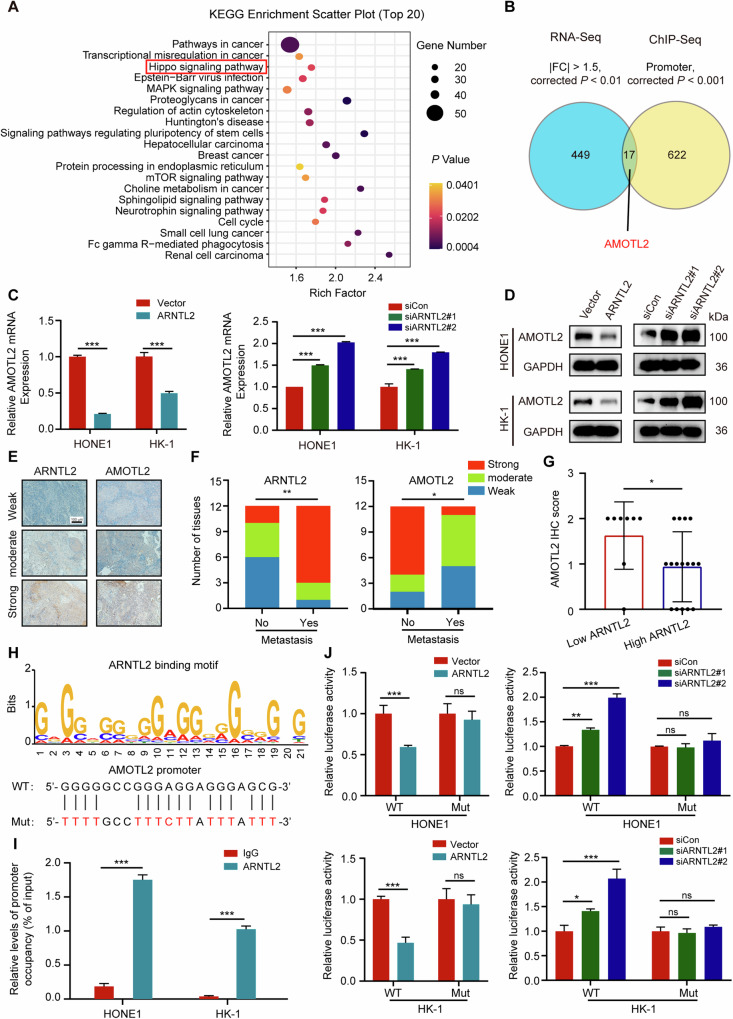
ARNTL2 suppresses the expression of AMOTL2 by regulating its transcription in NPC. **A** KEGG analysis based on the differentially expressed genes from the RNA-seq data. **B** Identification of potential targets of ARNTL2. 17 genes including AMOTL2 were obtained by overlapping the genes from the RNA-seq data ( | fold-change | > 1.5 and corrected *P* < 0.01) and the ChIP-seq data (promoter and corrected *P* < 0.001). **C**, **D** RT-q*P*CR and western blotting analyses of AMOTL2 expression in HONE1 and HK-1 cells with ARNTL2 overexpression or knockdown. **E** The staining extents of all sections were scored as follows: 0 (weak), 1 (moderate), and 2 (strong). Scale bar, 100 μm. **F** The IHC scores of AMOTL2 and ARNTL2 in NPC tissues without (n = 12) or with (n = 12) distant metastasis. **G** The IHC score of AMOTL2 in low ARNTL2 group and high ARNTL2 group in NPC tissues (n = 24). **H** ARNTL2-binding motif (up) and ARNTL2-binding site in AMOTL2 promoter predicted by ChIP-seq analysis. **I** ChIP-qPCR analysis of ARNTL2 at the AMOTL2 promoter in HONE1 and HK-1 cells transfected with ARNTL2 overexpression plasmid. **J** Luciferase reporter assays of AMOTL2 promoter activity in ARNTL2 overexpression or knockdown HONE1 and HK-1 cells with wild type or mutant luciferase reporter. The relative activity of cells was presented as firefly/renilla luciferase activity. Data are presented as means ± SD. *P < 0.05; **P < 0.01; ***P < 0.001; ns, not significant.

ARNTL2 is a basic helix-loop-helix (bHLH) transcription factor [16] and was correlated with transcriptional misregulation in cancer in KEGG pathway analysis based on the differential genes of the RNA-seq data (Fig. 3A), indicating a potential role of ARNTL2 in gene transcription in NPC progression. Thus, to gain a global view of ARNTL2-binding chromatin distribution, we conducted a ChIP-seq analysis in HONE1 cells with ARNTL2 overexpression. In total, we identified 1924 ARNTL2-binding peaks for 1612 genes (*P* < 0.001), among which 65.8% were localized to promoters (Supplementary Table S4). To further determine ARNTL2-regulated downstream target, we overlapped the genes from the RNA-seq data (|fold-change| > 1.5 and corrected *P* < 0.01) and the ChIP-seq data (promoter, corrected *P* < 0.001) and identified 17 overlapping genes (Fig. 3B and Supplementary Fig. S2). Among these, AMOTL2 attracted our interest. AMOTL2 is a member of angiomotin family that are initially identified as binding proteins of angiostatin that mediate endothelial cell migration and tube formation [17, 18]. Particularly, AMOTL2 is required for cell migration in zebrafish embryos and endothelial cells [19]. We found that the mRNA and protein levels of AMOTL2 were significantly changed regardless of ARNTL2 overexpression or knockdown in both HONE1 and HK-1 cells (Fig. 3C, D). Additionally, we examined the expression of ARNTL2 and AMOTL2 in 24 NPC tissues with or with distant metastasis using IHC staining and then performed IHC score for all sections according to the following criteria: 0 (weak), 1 (moderate), and 2 (strong) (Fig. 3E). ARNTL2 showed a higher expression in NPC tissues with metastasis, but AMOTL2 presented a opposite result (Fig. 3F). Subsequently, tissues with an ARNTL2 IHC score of 2 were classified as high expression group, low expression group otherwise. We found that low ARNTL2 group had a higher AMOTL2 expression (Fig. 3G), which indicated that the expression of AMOTL2 might be associated with NPC metastasis and was negatively regulated by ARNTL2 in NPC tissues.

To determine whether ARNTL2 represses AMOTL2 expression by regulating its transcription, we constructed AMOTL2 promoter WT and mutant plasmids (Fig. 3H and Supplementary Table S5), and ChIP-qPCR and dual-luciferase reporter assays were employed. As expected, ARNTL2 protein could directly bind to the promoter of AMOTL2 (Fig. 3I). Overexpression or knockdown of ARNTL2 markedly attenuated or enhanced the luciferase activity of AMOTL2 promoter, but failed to regulate the activity of the mutant AMOTL2 promoter (Fig. 3J), revealing that AMOTL2 was transcriptionally inhibited by ARNTL2.

### ARNTL2 increases YAP nuclear translocation by suppressing YAP phosphorylation

**Fig. 4:**
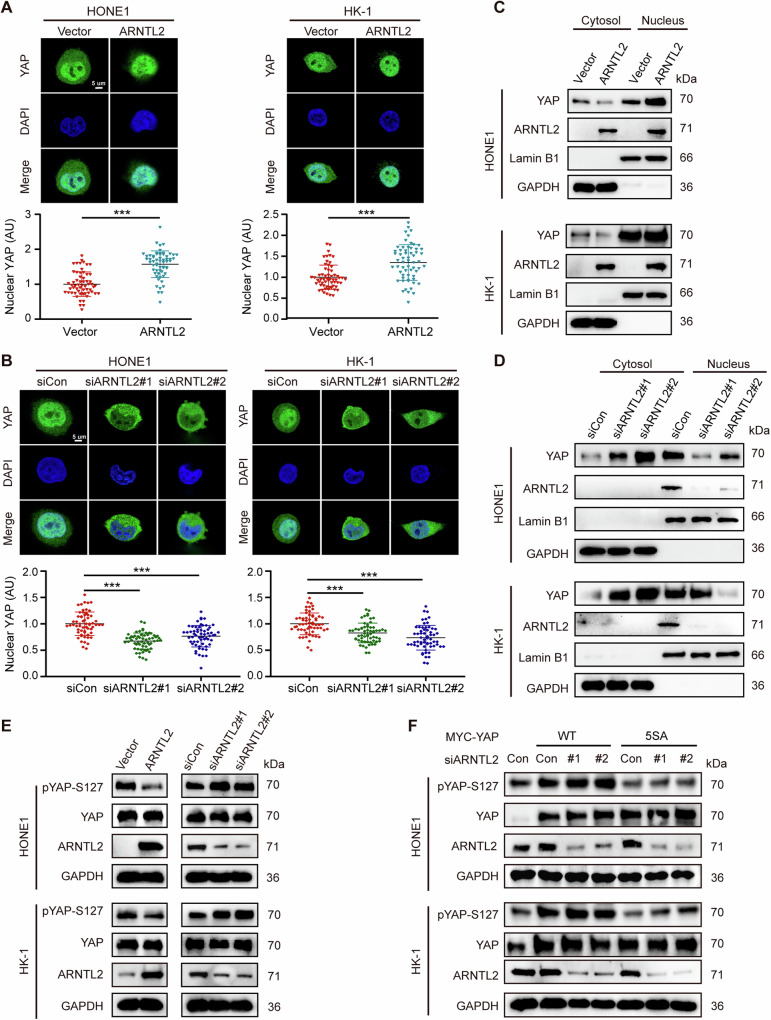
ARNTL2 increases YAP nuclear translocation by suppressing its phosphorylation. **A**, **B** Representative anti-YAP immunofluorescence images of HONE1 and HK-1 cells with ARNTL2 overexpression or knockdown (upper panel). The nuclear YAP intensities of 60 cells were calculated using Image J software (lower panel). Green, YAP; Blue, DAPI, Scale bar, 5 μm. **C**, **D** Western blotting for YAP protein from the cytosolic and nuclear extracts of HONE1 and HK-1 cells with ARNTL2 overexpression or knockdown. **E** Western blotting for pYAP-S127 and YAP proteins from HONE1 and HK-1 cells with ARNTL2 overexpression or knockdown. **F** Western blotting for pYAP-S127 and YAP proteins from ARNTL2-depletion HONE1 and HK-1 cells co-transfected with YAP-WT or YAP-5SA plasmid. Data are presented as means ± SD. ***P < 0.001.

KEGG pathway analysis demonstrated that ARNTL2 was associated with the Hippo signaling pathway (Fig. 3A). Hippo signaling pathway represents a tumor suppressor pathway that functions in diverse biological processes, including metastasis [20]. YAP is a key downstream effector of the Hippo signaling pathway, and its phosphorylation, as reported, drives cytoplasmic localization and prevents YAP-mediated transactivation in the nucleus [21]. Immunofluorescence assay showed that elevated ARNTL2 expression reduced the cytoplasmic localization, but augmented YAP nuclear localization, while silencing of ARNTL2 presented the opposite effect (Fig. 4A, B). The cytoplasmic and nuclear protein fractionation assay also exhibited consistent results (Fig. 4C, D). Studies have demonstrated the major mechanism of YAP excluded from the nucleus is regulated, to a large extent, by pYAP-S127 [22]. Thus, we detected the expression of pYAP-S127 and total YAP in HONE1 and HK-1 cells with ARNTL2 overexpression or knockdown. The results showed that ARNTL2 overexpression suppressed the expression of pYAP-S127, while ARNTL2 knockdown markedly facilitated the expression of pYAP-S127. Nevertheless, total YAP protein was not evidently changed regardless of overexpression or knockdown of ARNTL2 (Fig. 4E). Furthermore, we examined the impact of ARNTL2 knockdown on YAP phosphorylation in HONE and HK-1 cells expressing YAP WT or YAP 5SA mutant, which cannot be phosphorylated by LATS kinase. As expected, ARNTL2 depletion increased pYAP-S127 expression in YAP-WT-expressing cells, but had no evident effect in YAP 5SA-expressing cells (Fig. 4F), suggesting that ARNTL2 induces YAP translocation from the cytoplasm to the nucleus by regulating YAP phosphorylation.

### AMOTL2 facilitates LATS-dependent YAP phosphorylation via the recruitment and stabilization of LATS1/2 kinases

**Fig. 5:**
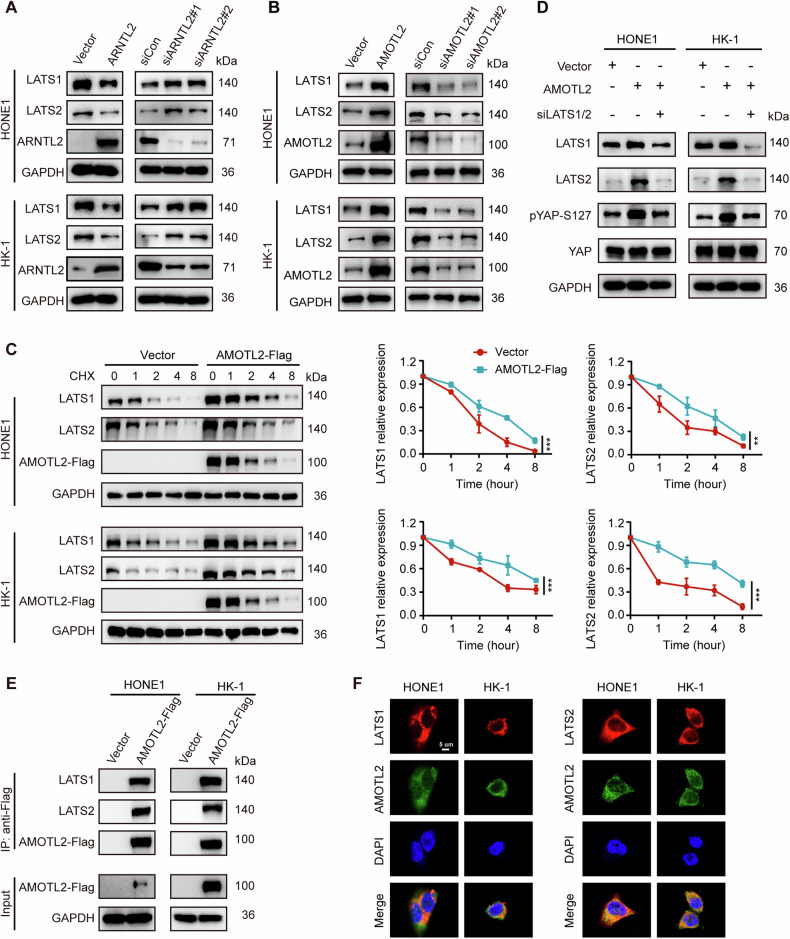
AMOTL2 facilitates LATS-dependent YAP phosphorylation via the recruitment and stabilization of LATS1/2 kinases. **A** Western blotting for LATS1/2 kinases in HONE1 and HK-1 cells with ARNTL2 overexpression or knockdown. **B** Western blotting for LATS1/2 kinases in HONE1 and HK-1 cells with AMOTL2 overexpression or knockdown. **C** Western blotting for LATS1/2 kinases in HONE1 and HK-1 cells transfected with AMOTL2-Flag and the empty vector plasmids after cycloheximide (CHX) treatment. **D** Western blotting for LATS1/2, pYAP-S127, and YAP proteins from HONE1 and HK-1 cells expressing AMOTL2 or vector and co-transfected with siLATS1/2. **E** Co-immunoprecipitation with anti-Flag antibody in HONE1 and HK-1 cells transfected with AMOTL2-Flag and the empty vector plasmids. **F** Immunofluorescence assays for colocalization between AMOTL2 and LATS1 or LATS2 in HONE1 and HK-1 cells. Red, LATS1 or LATS2; Green, AMOTL2; Blue, DAPI. Scale bar, 5. Data are presented as means ± SD. **P < 0.01, ***P < 0.001.

pYAP-S127 is a direct phosphorylation site by LATS kinase and is often used to gauge LATS activity [23]. Studies have demonstrated that AMOT family can recruit LATS1/2 kinases to influence their activities [24, 25]. Thus, we separately examined the effects of ARNTL2 and AMOTL2 after overexpression or knockdown on LATS1/2 expression, and found that ARNTL2 negatively regulate the expression of LATS1/2 (Fig. 5A) and AMOTL2 positively regulated the expression of LATS1/2 kinases (Fig. 5B). Furthermore, we also detected the LATS1/2 expression in HONE1 and HK-1 cells with AMOTL2 overexpression after cycloheximide (CHX) treatment. The results showed that AMOTL2 enhanced the stability of the endogenous and exogenous LATS1 and LATS2 kinases through CHX treatment (Fig. 5C). Notably, LATS1/2 silencing reversed AMOTL2-mediated upregulation of pYAP-S127 (Fig. 5D). Then, we performed co-immunoprecipitation and immunofluorescence assays to explore the interaction between AMOTL2 and LATS1/2. The results showed that AMOTL2 could bind to LATS1/2 kinases (Fig. 5E, F). These results suggest that AMOTL2 can recruit and stabilize LATS1/2 kinases.

### AMOTL2 is required for ARNTL2 facilitating NPC cell migration and invasion in vitro

**Fig. 6:**
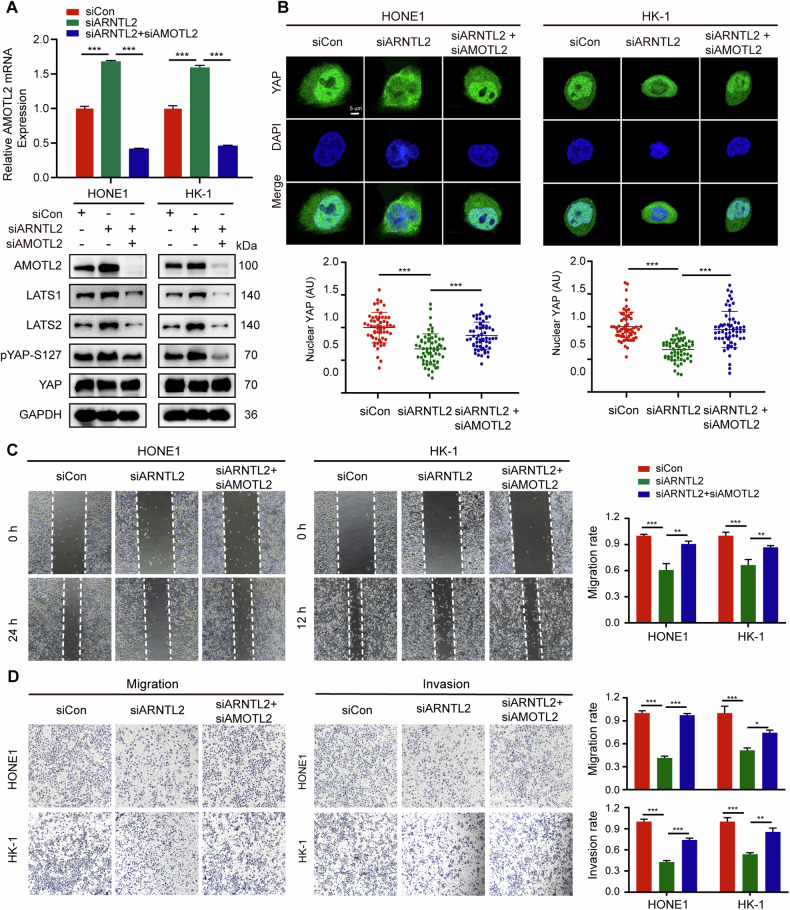
AMOTL2 is required for ARNTL2 promoting NPC cell migration and invasion abilities. **A** RT-qPCR for AMOTL2 and western blotting for AMOTL2, LATS1/2, pYAP-S127, and YAP proteins from HONE1 and HK-1 cells with concurrent knockdown of ARNTL2 and AMOTL2. **B** Representative anti-YAP immunofluorescence images of HONE1 and HK-1 cells with concurrent knockdown of ARNTL2 and AMOTL2 (upper panel). The nuclear YAP intensities of 60 cells were calculated using Image J software (lower panel). Green, YAP; Blue, DAPI. Scale bar, 5 μm. **C** Wound healing migration assay in HONE1 and HK-1 cells with concurrent knockdown of ARNTL2 and AMOTL2 (×100). **D** Transwell migration and invasion assays without or with Matrigel in HONE1 and HK-1 cells with concurrent knockdown of ARNTL2 and AMOTL2 (×100). Data are shown as means ± SD. *P < 0.05; **P < 0.01; ***P < 0.001.

As described in previous studies, AMOT family proteins including AMOTL2 exert a negative effect on YAP nuclear translocation [26], which is associated with tumor metastasis [20]. To investigate the importance of AMOTL2 in ARNTL2 regulating LATS-YAP pathway and NPC metastasis, we performed double-knockdown of ARNTL2 and AMOTL2 in HONE1 and HK-1 cells. The results showed that AMOTL2 knockdown reversed ARNTL2 inhibition-induced upregulation of AMOTL2, LATS1/2 and pYAP-S127 and downregulation of nuclear YAP (Fig. 6A, B), which revealed that AMOTL2 is required for the activation of LATS-YAP pathway by ARNTL2. Moreover, downregulation of AMOTL2 effectively rescued the inhibitory effect on NPC cell migration and invasion abilities mediated by ARNTL2 depletion (Fig. 6C, D). Based on these data, we propose that AMOTL2 is necessary for ARNTL2-driven YAP transporting to nucleus in LATS-dependent YAP phosphorylation manner, thus promoting NPC cell migration and invasion abilities.

### ARNTL2 knockdown disturbs NPC invasiveness and metastasis in vivo

**Fig. 7:**
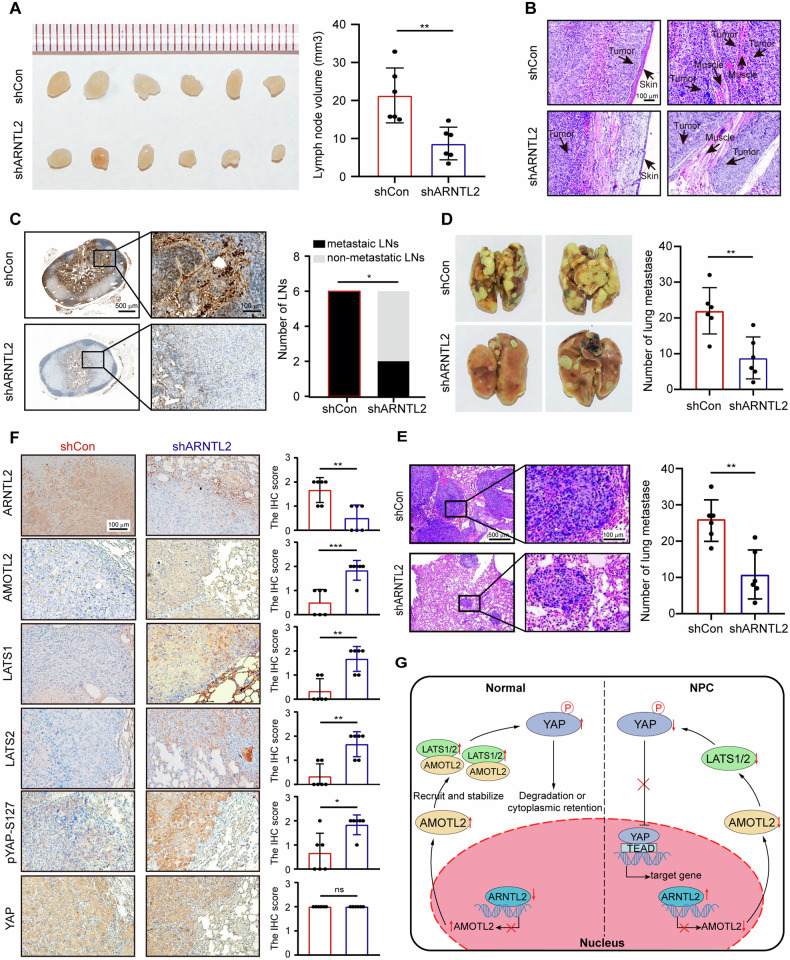
ARNTL2 knockdown disturbs NPC invasiveness and metastasis in vivo. Inguinal lymph node and lung metastasis models were constructed by injecting the stably ARNTL2-interfered HONE1 cells or control cells into the footpad or tail vein of nude mice (n = 6 per group). **A** Inguinal lymph nodes (left) and their volumes (right). volume = (length × width^2^)/2. **B** Representative images of H&E staining showed that the primary footpad tumor invaded in the skin and muscle. Scale bar, 100 μm. **C** Representative IHC images of pan-cytokeratin-positive inguinal lymph node (left) and the number of metastatic lymph nodes in the shARNTL2 and control groups. **D** Representative images of metastatic lung tumors and the number of metastatic nodules on the lung surface between the shARNTL2 and control groups. **E** Representative images of H&E staining for lung tumors and the number of microscopic metastatic nodules in the shARNTL2 and control groups. **F** Representative IHC images and IHC score for ARNTL2, AMOTL2, LATS1/2, pYAP-S127, and YAP expression in the lung metastases of the shARNTL2 and control groups. The intensities of staining were scored as 0 (weak), 1 (moderate), and 2 (strong). Scale bar, 100 μm. **G** A schematic model for the regulation of YAP by ARNTL2. Data are shown as means ± SD. *P < 0.05; **P < 0.01; ***P < 0.001; ns not significant .

To further confirm the functional importance of ARNTL2 in NPC metastasis in vivo, we constructed xenograft tumor metastasis models by transplanting stable ARNTL2-silenced HONE1 cells or corresponding control. Notably, the inguinal lymph node metastasis model revealed that the sizes and volumes of inguinal lymph nodes from nude mice in the shARNTL2 group were significantly smaller than those in the control group (Fig. 7A). Moreover, H&E staining of the footpad primary tumor showed that ARNTL2 knockdown caused a less aggressive phenotype toward the skin and muscle of the primary tumor (Fig. 7B). IHC staining of inguinal lymph nodes presented that the ratio of pan-CK-positive lymph nodes in the shARNTL2 group was markedly lower (Fig. 7C). Furthermore, to investigate the impact of ARNTL2 on distant metastasis of NPC, we established a lung metastasis model and observed that the metastatic nodules on the lung surfaces were significantly fewer in the shARNTL2 group than in the control group (Fig. 7D), and H&E staining also presented consistent result (Fig. 7E). IHC staining showed that the expression of AMOTL2, LATS1/2, and pYAP-S127 was markedly increased in the shARNTL2 group, while the expression of total YAP was not significantly changed in the two groups (Fig. 7F). Furthermore, despite the lack of evident effect on cell proliferation observed in vitro, we proceeded to establish xenograft tumor growth models, taking into consideration the complicated in vivo physiological environment. Intriguingly, the shARNTL2 group exhibited a noteworthy inhibition in tumor growth compared to the control group (Supplementary Fig. S3). These findings indicate that ARNTL2 promotes NPC lymphatic and pulmonary metastasis, along with tumor growth, by the inhibition of AMOTL2 and LATS-dependent YAP phosphorylation in vivo.

## Discussion

This present study highlights the significant role of the core circadian gene ARNTL2 in NPC invasiveness and metastasis both in vitro and in vivo. Here, we determined that the expression of ARNTL2, differing from ARNTL [15], was upregulated in both NPC tissues and cells. AMOTL2, a member of AMOT family, was identified as a new downstream of ARNTL2. ARNTL2 transcriptionally inhibited the expression of AMOTL2 to decrease the recruitment and stabilization of AMOTL2 to LATS1/2 kinases, ultimately leading to YAP nuclear translocation and facilitating NPC cell metastasis.

Recent studies have demonstrated that abnormal expression of circadian genes is known to engage in tumor initiation and progression through disrupting rhythms of cellular processes, such as nutrient metabolism, autophagy, DNA damage repair, redox regulation, protein folding, cellular secretion, and so on [27], thereby creating a conducive cellular microenvironment for tumorigenesis. Moreover, extensive in vitro and in vivo studies demonstrate that dysregulation of multiple core circadian genes is implicated in tumor development and treatment [14, 28–31]. Particularly, among circadian genes, ARNTL2, a core component of the circadian clock, has been demonstrated as a potent oncogene that facilitates various tumor invasiveness and metastasis via diverse molecular mechanisms. For instance, ARNTL2 drives lung adenocarcinoma metastatic self-sufficiency by orchestrating the expression of a complex pro-metastatic secretome [31]. In colorectal cancer, ARNTL2 is identified as a potential biomarker for tumor invasiveness and aggressiveness and ARNTL2 knockdown suppresses cell migration, invasion, and proliferation through inhibiting SMOC2-EMT expression and PI3K/AKT signaling pathway [32, 33]. Similarly, ARNTL2 also promotes pancreatic ductal adenocarcinoma progression through TGF/BETA pathway [34]. These studies collectively demonstrate that ARNTL2 is a pro-metastatic regulator of cancer. Consistent with the above findings, our study also revealed that ARNTL2 was highly expressed in NPC tissues and cells and substantially promoted NPC cell migration and invasion abilities in vitro and in vivo. Moreover, we found that ARNTL2 did not evidently affect NPC cell proliferation in vitro, but promoted tumor growth in vivo, which suggests that in vitro experiment may not completely mimic the complicated physiological conditions in vivo.

AMOTL2 is known to interrupt angiogenesis by suppressing endothelial cell proliferation and migration and disrupting cell polarity [17, 35, 36]. Studies demonstrate that restrained AMOTL2 expression induces the changes in cell morphology and epithelial-mesenchymal transition (EMT) in breast cells [26, 37]. Moreover, low expression of AMOTL2 facilitates glioblastoma proliferation and metastasis through regulating β‑catenin nuclear localization [36]. Nonetheless, some studies provide contradictory results. In colon cancer, AMOTL2 functions as an oncogene that promotes cell growth and invasion by disrupting the apical-basal polarity [38]. A potential explanation is that AMOTL2 includes two isoforms of p60 and p100, and the short isoform of AMOTL2 p60 for an oncogenic role is in contrast with the long isoform of AMOTL2 p100 for tumor suppression [38]. Additionally, AMOTL2 has been widely reported as a suppressor of the Hippo/YAP signaling pathway [39–41]. Depletion of AMOTL2 increases mouse liver size and activation of YAP [41]. AMOTL2 overexpression inhibits YAP-induced transcription, growth, and metastasis in multiple cancers [42]. In this study, we observed that the expression of AMOTL2 was significantly higher in NPC tissues with distant metastasis, suggesting the tight correlation with NPC metastasis. Moreover, we also found AMOTL2 could recruit and stabilize LATS1/2 kinases to promote YAP phosphorylation, which was consistent with previous studies [24, 25].

The present study has several limitations. First, only two NPC cell lines were used in the in vitro assays. Nevertheless, the pathogenesis of NPC is closely correlated with Epstein-Barr virus (EBV) infection [1]. EBV-negative NPC patients have poorer prognoses in clinical practice [43]. Thus, an EBV-negative NPC cell line (HONE1) and an EBV-positive NPC cell line (HK-1) have relatively good representativity. Moreover, the exact mechanism how AMOTL2 stabilizes LATS1/2 kinase was not be detailly explored in this study. Despite these limitations, this study is the first to elucidate the function and mechanism of ARNTL2 in NPC development.

In summary, Fig. 7G presents our schematic model for the regulation of YAP by ARNTL2. In NPC cells, the upregulation of ARNTL2 suppressed the transcription expression of AMOTL2, decreasing the recruitment and stabilization of AMOTL2 to LATS1/2 kinases, ultimately promoting YAP translocation to the nucleus and driving NPC invasiveness and metastasis. These findings further shed light on the molecular mechanism underlying NPC metastasis and give evidence for targeting ARNTL2 for future cancer therapy.

## Materials and methods

### Dataset acquisition and clinical specimens

Four quantification matrices of NPC and NPE tissues were downloaded from the Gene Expression Omnibus (GEO) database (accession numbers: GSE12452, GSE53819, GSE13597, and GSE61218). Additionally, 8 freshly frozen human NPE and NPC tissues and 24 paraffin-embedded NPC specimens with or without distant metastasis were obtained from Sun Yat-sen University Cancer Center (SYSUCC; China). This study was performed in accordance with the Declaration of Helsinki and approved by the Institutional Ethical Review Board of SYSUCC (approval number: B2024-227), and the written informed consents were obtained from patients.

### Cell lines and culture

Human NPE cell line (NP69) and NPC cell lines (CNE1, CNE2, HNE1, HK-1, HONE1, SUNE1) were kindly gifted by Professor Musheng Zeng from SYSUCC. HEK293T cell line was purchased from the American Type Culture Collection (ATCC, USA). All cell lines were routinely tested to ensure the absence of mycoplasma contamination using MycoBlue Mycoplasma Detector (#D101-02, Vazyme, Nanjing, China). NP69 cell was cultured in a keratinocyte serum-free medium (#17005042, ThermoFisher, Waltham, MA, USA) with bovine pituitary extract. NPC cells and HEK293 cells were maintained in RPMI-1640 or DMEM medium (Gibco, Billings, MT, USA) supplemented with 10% fetal bovine serum (FBS; ExCell Bio, Shanghai, China). All cells were kept in a humidified incubator set at 37 °C with 5% CO_2_.

### Reverse transcription (RT) and quantitative real-time PCR (qPCR)

According to the manufacturer’s instructions, total RNA from cells was isolated using an RNA Quick Purification kit (#RN001, EScience, Shanghai, China) and then reverse transcribed to cDNA by GoScript Reverse Transcription System (#A5001, Promega, Madison, WI, USA). Subsequently, qPCR reactions were performed using ChamQ SYBR qPCR Master Mix (#Q311-02, Vazyme). GAPDH was used as the endogenous control. Relative expression of genes was calculated as described previously [44, 45]. The specific primer sequences for qPCR are listed in Supplementary Table S1.

### Plasmids, small interfering RNA (siRNA), and transfection

According to the standard molecular methods, we constructed pSin-EF2-ARNTL2-Flag-puro, pcDNA3.1-AMOTL2-3×Flag, pCMV-YAP-3×Myc-Neo, and pCMV-YAP 5SA-3×Myc-Neo plasmids. The siRNAs were purchased from RiboBio (RiboBio, Guangzhou, China), and their sequences were presented in Supplementary Table S2. The sequence of siARNTL2#1 was cloned into pLKO.1 plasmid to obtain shARNTL2. Moreover, ARNTL2-binding AMOTL2 promotor region (500 bp) predicted by ChIP-seq analysis and the indicated mutant were cloned into the pGL3-basic vectors.

NPC cells were transiently transfected with plasmids using Neofect^TM^ DNA transfection reagent (#TF20121201, Neofect, Beijing, China) and transfected with siRNA using Lipofectamine 3000 reagent (#L3000015, Invitrogen, Carlsbad, CA, USA).

### RNA sequencing (RNA-seq) and bioinformatic analysis

Total RNA from HONE1 cells transfected with siARNTL2#1 or siCon was extracted using a TRIzol reagent kit (#15596018, Invitrogen). RNA sequencing libraries were constructed using the NEBNext® UltraTM RNA Library Prep Kit for Illumina® (#E7530L, NEB, Ipswich, Massachusetts, USA). The sequencing was carried out on an Illumina Novaseq 6000 platform and 150 bp paired-end reads were generated. Reads were mapped onto a human reference genome (assembly GRCh38) by using HISAT2 (v 2.0.4). Fragments per kilobase of exon million mapped reads (FPKM) were calculated to evaluate gene abundance. The differentially expressed genes with |fold change| > 1.5 and *P* < 0.05 were considered significant. Heatmap was generated using R software (v3.4.3). The significant pathways of the differential genes were investigated using Kyoto Encyclopedia of Genes and Genomes pathway analysis (KEGG, http://www.genome.ad.jp/kegg). The potential functions of ARNTL2 were identified using Gene set enrichment analysis (GSEA) with gene sets downloaded from the molecular signature database (MSigDB, v.7.2, http://www.broadinstitute.org/gsea/msigdb).

### Nuclear and cytoplasmic protein fractionation

The nuclear and cytoplasmic proteins were separated using NE-PER nuclear and cytoplasmic extraction kit following the manufacturer’s instruction (#78833, ThermoFisher). The extracted proteins were subsequently used for western blotting analysis. Lamin B1 and GAPDH were used as the nuclear and cytoplasmic controls, respectively.

### Western blotting

Total protein from cells was extracted using RIPA buffer (#P0013B, Beyotime, Shanghai, China) containing EDTA-free protease inhibitor (#4693159001, Roche, Basel, Switzerland) and phosphatase inhibitor (#4906837001, Roche). Equal amounts of total proteins were subjected to electrophoresis with a 10% PAGE Gel Fast Preparation Kit (#PG112, EpiZyme, Shanghai, China) and then transblotted to 0.45 μm polyvinylidene fluoride (PVDF) membranes (#IPVH00010, Merck Millipore, Billerica, MA, USA). The membranes were blocked for 30 min with 5% non-fat powdered milk (#A600669-0250, Sangon Biotech, Shanghai, China), and then incubated with primary antibodies overnight at 4 °C and secondary antibodies for 1 h at room temperature. The primary antibodies used were presented as follows: anti-ARNTL2 (#sc365469, 1:50, Santa Cruz, USA), anti-AMOTL2 (#23351-1-AP, 1:1000, ProteinTech, Wuhan, China), anti-YAP (#13584-1-AP, 1:2000, ProteinTech), anti-pYAP-S127 (YAP phosphorylation site serine 127; #13008, 1:1000, Cell Signaling Technology, CST, Beverly, MA, USA), anti-LATS1 (#C66B5, 1:1000, CST), anti-LATS2 (#D83D6, 1:1000, CST), anti-Lamin B1 (#ab133741, 1:1000, Abcam, Cambridge, UK), anti-GAPDH (#ab8245, 1:5000, Abcam), and anti-Flag M2 (#F1804, 1:1000, Sigma, Carlsbad, CA, USA) antibodies.

### Lentivirus packaging and stable cell line construction

As previously described [46], HEK293T cells at a 60–80% confluence were co-transfected with PLKO.1-shARNTL2 or PLKO.1-shCon plasmid and lentiviral packaging plasmids (pMD.2G and psPAX2) using polyethyleneimine (#40816ES03, Yeasen, Shanghai, China). The virus-containing cell supernatant was harvested to infect HONE1 cells after 48 h. Subsequently, cells were screened with 2 μg/ml puromycin for at least 4–5 passages. RT-qPCR and western blotting were performed to examine the knockdown efficiency of ARNTL2 in the stable cell lines.

### Co-immunoprecipitation

NPC cells with Flag-AMOTL2 overexpression were harvested and lysed using IP lysis buffer (#87788, ThermoFisher) containing protease inhibitor (Roche). After centrifugation, the supernatants were incubated with anti-Flag antibody-conjugated magnetic beads (#M8823, Sigma-Aldrich) at 4 °C overnight. The beads were then washed and subjected to western blotting.

### Immunohistochemistry (IHC)

IHC was carried out on paraffin-embedded sections as previously described [47]. The primary antibodies were as follows: anti-ARNTL2 (1:50), anti-AMOTL2 (1:200), anti-YAP (1:100), anti-pYAP-S127 (1:200), anti-LATS1 (1:200), anti-LATS2 (1:200), and pan-cytokeratin (pan-CK; #ab234297, 1:1000; Abcam). The staining extents of all sections were scored as follows: 0 (weak), 1 (moderate), and 2 (strong).

### Immunofluorescence

NPC cells (5 × 10^4^ cells/ well) were seeded on coverslips in 24-well plates. The adherent cells were fixed with 4% paraformaldehyde for 30 min, permeabilized with 0.5% Triton X-100 for 20 min, and blocked with 1% bovine serum albumin (BSA) for at least 30 min. Subsequently, the cells were incubated with primary antibody against YAP (1:50), AMOTL2 (1:500), LATS1 (#17049-1-AP, 1:50, ProteinTech), and LATS2 (#20276-1-AP, 1:50, ProteinTech) overnight at 4 °C and Alexa FluorTM 488 goat anti-rabbit or 647 goat anti-mouse IgG secondary antibodies (1:1000, Invitrogen) for 1 h at room temperature. Cell nuclei were counterstained with Hoechst (#H3570, 1:500, Invitrogen) for 3 min. Images were acquired using a confocal laser-scanning microscope (Zeiss, LSM-980, Germany).

### Wound healing assay

HONE1 or HK-1 cells were transfected for 24 h and replaced with an FBS-free medium until near confluence in 6-well plates. Subsequently, cells were scratched using a 200 μl pipette tip. Images (100×) were captured at 0 h and 24 h (HONE1) or 12 h (HK-1) using an inverted microscope (Leica, Germany).

### Transwell migration and invasion assays

HONE1 (5 × 10^4^ or 1 × 10^5^) or HK-1 (1 × 10^5^ or 2 × 10^5^) cells were suspended in 200 μl FBS-free medium and added into the upper Transwell chambers (8-μm pores, Corning, NY, USA) without matrigel for migration assay or with matrigel (1:9; #356237, Corning) for invasion assay, while 500 μl medium containing 10% FBS was plated into the lower chambers. After 12 h (HONE1) or 40 h (HK-1) incubation, cells migrating to the lower surface of the chamber were fixed with methyl alcohol for 30 min and then stained with crystal violet for 2 h. The chambers were captured at 100 × magnification using an inverted microscope (Leica) and counted using Image J (http://imagej.nih.gov/ij).

### Cell counting kit8 (CCK8) and colony formation assays

Cell proliferation ability was measured using CCK8 and colony formation assays. Briefly, for the CCK8 assay, 1 × 10^3^ cells were seeded into 96-well plates. Each day on 1–5 days of incubation, 10 μl of CCK8 (#CK04, Dojindo, Kumamoto, Japan) was added into each well, and the absorbance values were measured at 450 nm. For colony formation assay, 1 × 10^3^ cells were cultured in 6-well plates for 1-2 weeks. The colonies were fixed by methanol and stained by hematoxylin.

### Chromatin immunoprecipitation (ChIP) assay

ChIP assay was performed using a pierceTM Magnetic ChIP Kit (#26157, ThermoFisher) according to its protocol. Briefly, cells were crosslinked with 1% formaldehyde for 10 min and quenched with 125 mM glycine at room temperature. Then, the cell nuclei were isolated, lysed, and sonicated to yield 200–1000 bp chromatin fragments. The chromatin fragments were immunoprecipitated with 3 μl anti-HA antibody (#ab9110, Abcam) or anti-rabbit IgG antibody (#26157, ThermoFisher) overnight at 4 °C and then immobilized on protein A/G magnetic beads for 2 h at 4 °C. After washing, the enriched DNA was eluted, de-crosslinked, purified, and then subjected to ChIP-seq or qPCR analyses. The specific primers for ChIP-qPCR are displayed in Supplementary Table S1.

### ChIP-seq and data analysis

The obtained ChIP-DNA and input DNA fragments were first end-repaired and A-tailed using the NEBNext End Repair/dA-Tailing Module (#E7442, NEB) and then ligated adapter using the NEBNext Ultra Ligation Module (#E7445, NEB). The DNA libraries were amplified for 15 cycles and then subjected to high-throughput sequencing using Illumina NextSeq 500. Subsequently, Data analysis was constructed. Briefly, the sequencing adapters, short reads (length < 35 bp), and low-quality reads were removed to obtain high-quality clean reads, which were confirmed using FastQC. Then, the reads were aligned to a human genome (assembly GRCh38) using Bowtie2 software (v2.2.6). Peak detection was carried out using MACS (v2.1.1) and 0.01 was set as the cut-off value. The peak sites to gene features were annotated using the ChIP seeker R package.

### Dual-luciferase reporter assay

NPC cells were co-transfected with ARNTL2 overexpressing plasmid or siARNTL2 (#1 and #2) or corresponding negative control, along with the pGL3-AMOTL2 wild type (WT) or pGL3-AMOTL2 mutant plasmid and a phRL-TK-Renella luciferase control vector. After 24 h, the luciferase activities of cells were detected using a Dual-Luciferase Reporter Assay System (#E1910, Promega). The relative activity of cells was presented as firefly/renilla luciferase activity.

### In vivo xenograft tumor models

BALB/c nude mice (female, 14–18 g, 4–5 weeks old) were purchased from Zhejiang Vital River Lab Animal Technology Co., Ltd (Zhejiang, China) for all xenograft assays. 2 × 10^5^ HONE1 cells stably expressing shARNTL2 were injected into the mice footpad for constructing the inguinal lymph node metastasis model (n = 6 per group), and 8 × 10^5^ cells were implanted into the tail vein of mice for lung metastatic model (n = 6 per group). Mice were observed every three days. After 4 weeks, these mice in the inguinal lymph node metastasis model were euthanized. The primary footpad tumors were dissected for Hematoxylin-eosin (H&E) staining, and the inguinal lymph nodes were collected for IHC staining targeting pan-CK. The lymph nodes with positive-pan-CK staining were considered metastatic. Moreover, lymph node volumes were calculated based on the formula of (length × width^2^)/2. After 5 weeks, these mice in the lung metastasis model were sacrificed. Lung metastases were counted and stained using H&E and IHC staining.

For the establishment of xenograft tumor growth model, 1 × 10^6^ HONE1 cells stably expressing shARNTL2 and control were subcutaneously injected into the armpit of mice (n = 6 per group). The tumor sizes of these mice were measured every three days, and tumor volume was calculated based on the formula of (length × width^2^)/2. After 18 days, these mice were euthanized and tumors were dissected and weighted.

All animal work complied with the National Institutes of Health Guide for the Care and Use of Laboratory Animals and has been approved by the Institutional Animal Care and Use Ethics Committee of SYSUCC (approval number: L0255202107006).

### Statistical analysis

All quantitative data were presented as the means ± standard deviation. The difference comparisons of the groups were performed using Student’s t-test or one-way ANOVA or two-way repeated-measures ANOVA with Bonferronis test. All statistical analyses were performed using SPSS software (version 26, IBM Corp), a two-tailed *P* value < 0.05 was considered significant.

### Supplementary information

Supplementary Figure and Table Legends

Supplementary Fig. S1

Supplementary Fig. S2

Supplementary Fig. S3

Supplementary Tables

Original western blots

## Data Availability

Raw data relevant to this study have been uploaded to the Research Data Deposit public platform (www.researchdata.org.cn). The RNA-seq and ChIP-seq data used in this study have been deposited in the Gene Expression Omnibus (GEO) database under the accession number GSE234440 and GSE234441.
