# Supplementary material for: The circadian gene ARNTL2 promotes nasopharyngeal carcinoma invasiveness and metastasis through suppressing AMOTL2-LATS-YAP pathway
Source: Cell Death Dis. 2024 Jul 2;15(7):466. doi: 10.1038/s41419-024-06860-x (PMC11220028; doi:10.1038/s41419-024-06860-x)
Supplement: Supplementary file 1 — Supplementary Figure and Table Legends [file 41419_2024_6860_MOESM1_ESM.docx]

Supplementary Materials

**Supplementary Figure Legends**

**Fig. S1 ARNTL2 did not evidently affect NPC cell proliferation *in vitro*.** (**A**) CCK8 assay in HONE1 and HK-1 cells with ARNTL2 overexpression or knockdown. (**B**) Representative images of colony formation assay in HONE1 and HK-1 cells with ARNTL2 overexpression or knockdown. Data are shown as means ± SD.

**Fig. S2 RT-qPCR analysis of 16 overlapped genes other than AMOTL2 in HONE1 and HK-1 cells.** 17 overlapped genes from the RNA-seq data (|fold-change| > 1.5 and corrected *P* < 0.01) and ChIP-seq data (promoter, corrected *P* < 0.001) were identified. The mRNA levels of 16 genes other than AMOTL2 were detected in HONE1 and HK-1 cells with ARNTL2 overexpression and knockdown using RT-qPCR. Data are shown as means ± SD.

**Fig. S3 ARNTL2 facilitated NPC xenograft tumor growth *in vivo*.** HONE1 cells stably expressing shARNTL2 and corresponding control were subcutaneously injected into the armpit of mice (n = 6 per group). (**A**) Photograph of xenograft of tumors. (**B**) The tumor growth curves of mice with ARNTL2 knockdown or control. Tumor volume was calculated based on the formula of (length × width^2^)/2. (**C**) The weights of tumors. Data are shown as means ± SD. ****P* < 0.001.

**Supplementary Table Legends**

**Supplementary Table S1.** Sequences of the qPCR primers and siRNA.

**Supplementary Table S2.** Sequences of the siRNA.

**Supplementary Table S3.** The differential genes in the RNA-seq.

**Supplementary Table S4.** The peaks of the ChIP-seq.

**Supplementary Table S5.** ARNTL2-binding sites on AMOTL2 promoter predicted by the ChIP-seq.
