# Supplementary figures and images for: The circadian gene ARNTL2 promotes nasopharyngeal carcinoma invasiveness and metastasis through suppressing AMOTL2-LATS-YAP pathway
Source: Cell Death Dis. 2024 Jul 2;15(7):466. doi: 10.1038/s41419-024-06860-x (PMC11220028; doi:10.1038/s41419-024-06860-x)

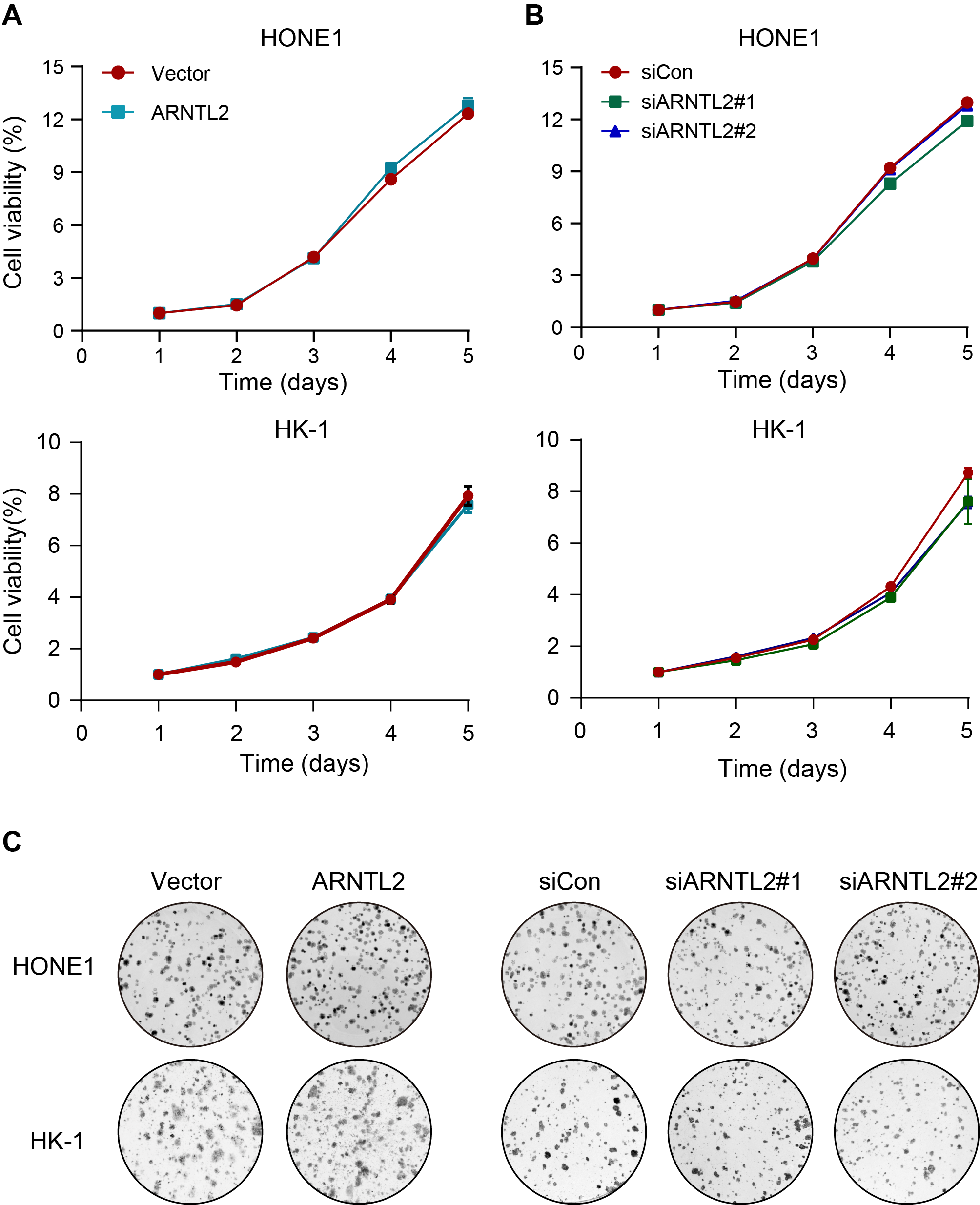

Supplement: Supplementary file 2 — Supplementary Fig. S1 [file 41419_2024_6860_MOESM2_ESM.tif]

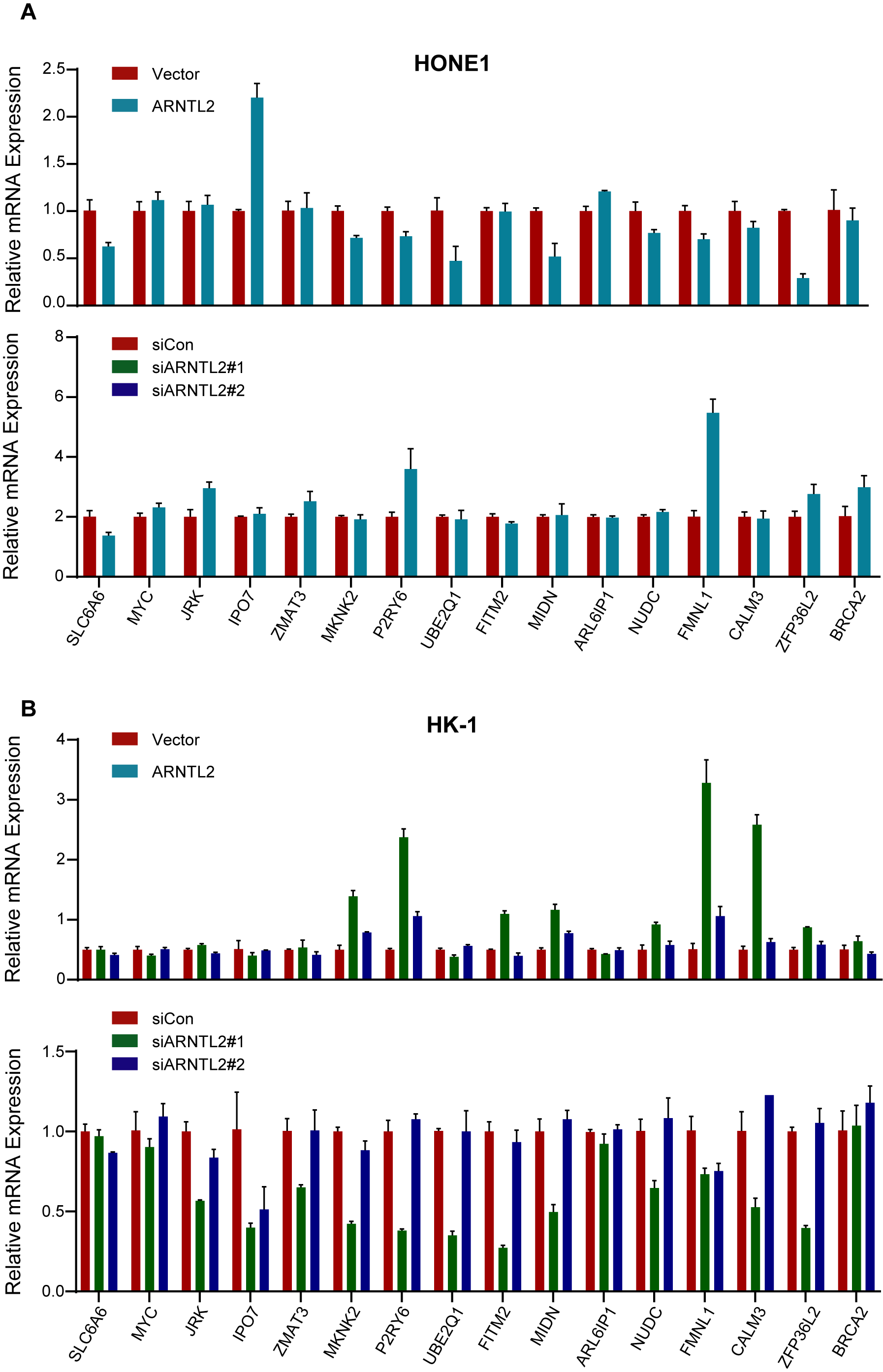

Supplement: Supplementary file 3 — Supplementary Fig. S2 [file 41419_2024_6860_MOESM3_ESM.tif]

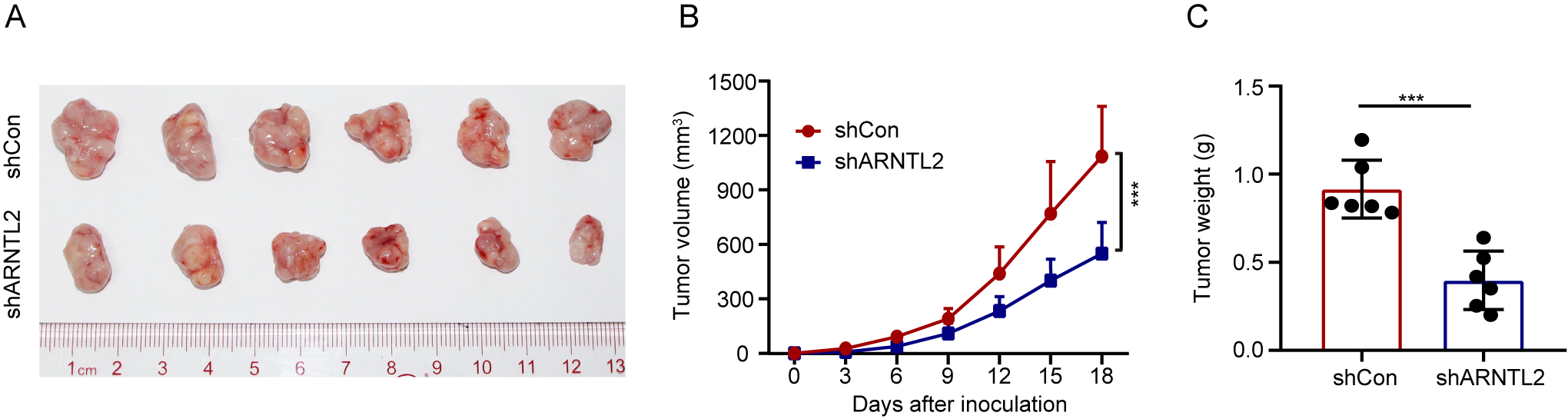

Supplement: Supplementary file 4 — Supplementary Fig. S3 [file 41419_2024_6860_MOESM4_ESM.tif]

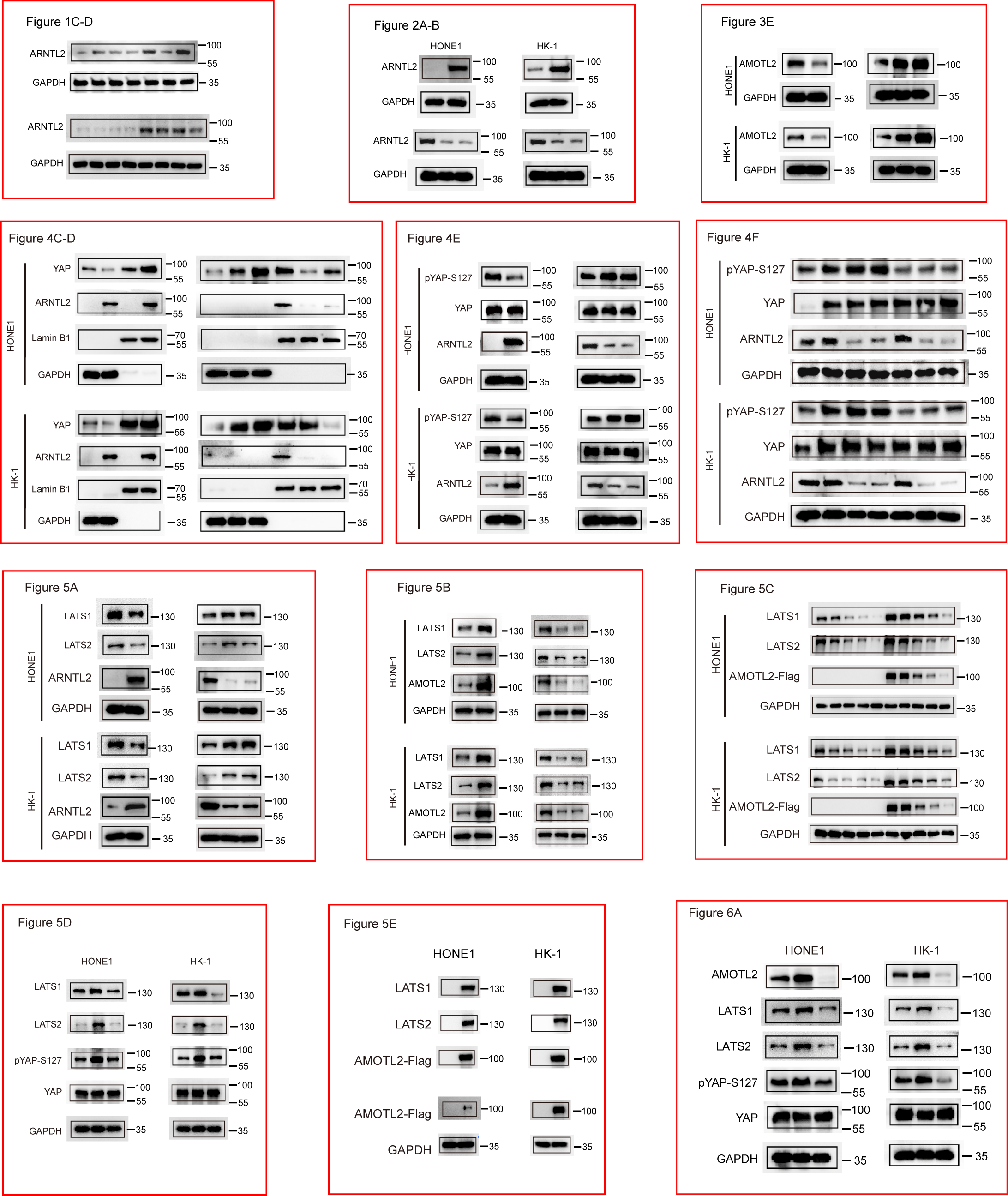

Supplement: Supplementary file 6 — Original western blots [file 41419_2024_6860_MOESM6_ESM.tif]
